# Menadione-Induced Oxidative Stress Re-Shapes the Oxylipin Profile of *Aspergillus flavus* and Its Lifestyle

**DOI:** 10.3390/toxins7104315

**Published:** 2015-10-23

**Authors:** Marco Zaccaria, Matteo Ludovici, Simona Marianna Sanzani, Antonio Ippolito, Riccardo Aiese Cigliano, Walter Sanseverino, Marzia Scarpari, Valeria Scala, Corrado Fanelli, Massimo Reverberi

**Affiliations:** 1 Department of Environmental Biology, University of Rome Sapienza, Largo Cristina di Svezia 24, Rome 00165, Italy; E-Mails: marco.zaccaria@uniroma1.it (M.Z.); matteo.ludovici@uniroma1.it (M.L.); marzia.scarpari@uniroma1.it (M.S.); valeria.scala@uniroma1.it (V.S.); corrado.fanelli@uniroma1.it (C.F.); 2 Department of Soil, Plant and Food Sciences, University of Bari Aldo Moro, Via Giovanni Amendola 165/A, Bari 70126, Italy; E-Mails: simonamarianna.sanzani@uniba.it (S.M.S.); antonio.ippolito@uniba.it (A.I.); 3 Sequentia Biotech SL, Calle Comte d’Urgell 240 3°D, Barcelona 08036, Spain; E-Mails: raiesecigliano@sequentiabiotech.com (R.A.C.); wsanseverino@sequentiabiotech.com (W.S.)

**Keywords:** oxylipins, oxidative stress, menadione, RNA-seq, LC-MS/MS

## Abstract

*Aspergillus flavus* is an efficient producer of mycotoxins, particularly aflatoxin B_1_, probably the most hepatocarcinogenic naturally-occurring compound. Although the inducing agents of toxin synthesis are not unanimously identified, there is evidence that oxidative stress is one of the main actors in play. In our study, we use menadione, a quinone extensively implemented in studies on ROS response in animal cells, for causing stress to *A. flavus.* For uncovering the molecular determinants that drive *A. flavus* in challenging oxidative stress conditions, we have evaluated a wide spectrum of several different parameters, ranging from metabolic (ROS and oxylipin profile) to transcriptional analysis (RNA-seq). There emerges a scenario in which *A. flavus* activates several metabolic processes under oxidative stress conditions for limiting the ROS-associated detrimental effects, as well as for triggering adaptive and escape strategies.

## 1. Introduction

*Aspergillus flavus* is a cosmopolitan pathogen of oily and starchy seeds. It is an efficient producer of mycotoxins, particularly aflatoxin B_1_. Although it is actually hard to pinpoint the environmental conditions inducing aflatoxin B_1_ synthesis, there is evidence that oxidative stress is one of the main actors in play [1,2,3].

Intracellular ROS (reactive oxygen species) concentration is high wherever oxygen is metabolically involved, particularly in mitochondria and peroxisomes. ROS accumulate mostly because of cellular respiration. They are by-products of the activity of the mitochondrion, detrimental to many of its own constituents: cell membrane, enzymes and DNA. Peroxisomal β-oxidation also leads to an intense production of ROS. In relation to this, fungal cells possess an extensive inventory of enzymes to keep the redox balance under control; most of these enzymes use H_2_O_2_ and O_2_^−^ as substrates [4]. In peroxisomes, H_2_O_2_ accumulation is in no small part a consequence of fatty acid β-oxidation. Since the early stages of the evolution of aerobic life, ROS have exerted a strong influence on several aspects of cell metabolism [5]. Over time, ROS have extended their role: from the original condition of stressing agents, to signaling molecules able to control major metabolic processes, such as development and resistance to stresses [6]. ROS have thus represented dominant driving forces in the evolution of animal, plant and fungal species alike [1,2,3,4,5,6,7,8]. The NADPH-dependent oxidase system is a complex responsible for the production of ROS as eliciting molecules, superoxide anion and H_2_O_2_ in particular [9]; the oxidative burst they elicit, *i.e.*, a self-induced spike in the concentration of intracellular ROS, often represents the first response to host invasion.

Several *in vitro*-based methods currently exist to induce oxidative stress, although cellular response is highly specific to the oxidant employed, as no single oxidant is representative of cell endogenous oxidative stress [10]. To gain keen insight into the metabolic implications of ROS and their transcriptomic premises, while keeping all of the practical advantages granted by *in vitro* experiments, the choice of the appropriate oxidative compound is of paramount importance. Menadione (2-methylnaphthalene-1,4-dione), a quinone and a precursor of vitamin K, is employed in several branches of biological research as a pro-oxidant agent [11]. Menadione acts as an apoptosis-inducing agent through elicitation of mitochondrial depolarization. Enzymes employing NAD(P)H as an electron donor are apt to metabolize quinones via one-electron reduction reactions, with an unstable semi-quinone as the outcome. When O_2_ is present, semi-quinones are involved in a redox cycle that regenerates the original quinone plus O_2_^−^ [12], progressively increasing the intracellular concentration of ROS.

Henceforth, the oxidative stress induced by menadione reproduces the metabolic signal selected throughout evolution to be upstream of many basic metabolic responses.

As previously observed [1], aflatoxin synthesis implies a boost in oxygen uptake (a 2.4-fold increase) always followed by an increase in oxidative stress owing to the consequential ROS generation. This increased rate of oxygen uptake stands at the turning point between trophophase (the active growth stage) and idiophase (when secondary metabolites are prevalently produced). Conversely, a switch towards fatty acid metabolism in lieu of glucose catabolism underlines the onset of idiophase; in *Aspergilli*, this is concomitant to the onset of sporulation, along with cellular development, differentiation, virulence and, in mycotoxigenic strains, such as *A. flavus*, aflatoxin synthesis [13]. Within fatty acid-derived molecules, oxidized fatty acids, *i.e.*, oxylipins, are cardinal to several of such aspects. Lipoperoxidation is one more way to increase intracellular ROS concentration [14]; an oxidative burst mediated in this way is usually quite intense and triggered at late developmental stages. In *Aspergillus flavus*, aflatoxin B_1_ synthesis is the metabolic response to fend off a late oxidative insult, whenever antioxidant enzymes are not up to the task [15].

In our study, we combine transcriptional and metabolic studies for giving indications of the relation between ROS and oxylipin formation, trying to elucidate how they may influence conidiation and secondary metabolism activation in *A. flavus*.

## 2. Materials and Methods

### 2.1. Fungal Strain and Culture Conditions

The strain we used is *Aspergillus flavus* NRRL 3357. An amount, 10^5^ conidia/mL, was inoculated in 50 mL of Czapek Dox Broth (Difco, Sparks, MD, USA) with the addition of NaMoO_4_ (1 mg/L) and ZnSO_4_ (5 mg/L) and kept at stationary growth conditions, in 100-mL Erlenmeyer flasks, 28 °C for 96 h. After this point, oxidative stress was induced by the addition of 0.1 mM menadione (crystalline menadione; Sigma-Aldrich, St. Louis, MO, USA), dissolved in dimethyl sulfoxide; growth parameters were kept unvaried. Mycelia were finally collected at different time intervals after menadione amendment (24, 48, 96, 168 h post-amendment (hpa)).

### 2.2. Quantification of Mycelial Growth, Conidiogenesis and Aflatoxin B_1_ Production

At each time interval (24, 48, 96, 168 hpa), mycelium weight was assayed after filtration (Millipore filters, Millipore, Darmstadt, Germany; 0.45 μm) and lyophilization; conidiogenesis was determined sampling 1 mL of culture medium after stirring the mycelium with the addition of 0.01% *w*/*v* Triton solution. Conidia concentration was estimated in the cell count chamber; aflatoxin B_1_ was extracted from 2-mL culture medium samples with chloroform: methanol (2:1 *v*/*v*), reduced under a N_2_ stream and quantified by HPLC analysis (Agilent Technologies, Santa Clara, CA, USA) [3].

### 2.3. Antioxidant Enzymes and Intracellular ROS Assays

Superoxide dismutase (SOD) and catalase (CAT) activity was estimated with a spectrophotometric assay, as previously described [2]. Intracellular ROS concentration was quantified from lyophilized mycelia (10 mg) with spectrofluorometric assays at each time interval. The concentration of O_2_^−^ and H_2_O_2_ was detected and referred to a standard curve as in Reverberi *et al.* [3]. Peroxynitrite (ONOO^−^ was assayed as described in Cao *et al.* [16].

### 2.4. Identification of Differentially-Expressed Genes via qRT-PCR

Extraction of RNA was performed on lyophilized mycelia, collected at the four different time intervals, employing the TRI-REAGENT method (Sigma-Aldrich) with the addition of a chloroform:isoamylic (24:1) purification step. Complementary DNA was synthesized with the “1st Strand cDNA synthesis SUPER SCRIPT II for RT-PCR” kit (Invitrogen, Carlsbad, CA, USA) and then used for qRT-PCR analysis. The SYBR No-ROX Kit (SensiMix, London, UK) was used. Samples were prepared in triplicates of a 20-μL reaction mixture. Amplification was carried out with LineGene K PCR detection systems (Bioer, Hangzhou, China) with the following cycling conditions: 1 denaturation step of 10 min long (95 °C); 50 cycles comprising: (1) a 15-s step for denaturation at 95 °C; (2) a 20-s step for annealing at the temperature recommended by the primer manufacturer (Sigma-Aldrich); (3) a 30-s step for elongation (73 °C). Genes of interest are markers for the Krebs cycle (aconitase, succinate dehydrogenase), the pentose phosphate pathway (transaldolase, glucose 6-phosphate dehydrogenase) and oxidative stress response (*AP-1*, *AtfB*). β-tubulin was used as the housekeeping gene. Primer sequences are provided in Table S1.

### 2.5. RNA-seq Analysis

Six cDNA libraries were prepared, representing the transcriptome of *A. flavus* during the presence or absence of oxidative stress. All of the experiments were conducted at three different time points (24, 48 and 96 hpa). Libraries were prepared using the TruSeq™ RNA sample preparation kit (Illumina, San Diego, CA, USA) from 4 μg of total RNA (low-throughput protocol), according to the manufacturer’s instructions. Thereafter, the obtained cDNA was subjected to electrophoresis on 1% agarose gel and size-checked at approximately 240 bp. Libraries were quantified by spectrophotometry (NanoDrop 2000, Wilmington, DE, USA) and a fluorimeter (Qubit 2.0, Life-Technologies, Carlsbad, CA, USA), pooled, normalized at a concentration of 10 pM (as suggested by the manufacturer) and loaded into the flow-cell. Cluster generation was performed in an Illumina cluster station (cBot). Sequence runs of 50 cycles were carried out on a HiScanSQ (Illumina). Base calling was performed using Illumina pipeline software CASAVA 1.8.1. In this way, sequence reads with a length of 50 bp were acquired. The reads underwent quality control, and adaptor or contaminant sequences were removed. Sequencing produced 15.8–16.9 million reads per library, 94% of which was above the quality score of 30. The raw sequence data were uploaded on the Server Root Administrator server (Accession Number PRJNA294865). Raw sequences were processed, filtered and normalized using the Illumina pipeline to generate fast-q files. All reads were mapped with Tophat 2.0.11 software to *A. flavus* coding sequences, to calculate, by using edgeR software (Fred Hutchinson Cancer Research Center, Seattle, WA, USA), the RPKM (reads per kilobase of transcript per million mapped reads) for each clustered gene. Multiple gene lists were generated corresponding to different RPKM-based comparisons between stress conditions and sampling times, ultimately enabling the identification of new and known differentially-expressed genes. Protein interaction networks were then built. Raw reads were checked for quality with FastQC v0.10.1, and then, trimming and removal of adapters were performed with Trimmomatic v0.33 [17]. The obtained reads were then mapped against the *Aspergillus flavus* genome (JCVI-afl1-v2.0.23. NSDC Assembly GCA_000006275.1) with TopHat v2.0.11 [18], providing the reference gene annotation file with known transcripts. FeatureCounts [19] was used to perform read summarization at the gene level and default options. Differential expression analysis was performed with R using the package edgeR [20]. Gene ontology enrichment analysis was conducted with the software agriGO (Beijing, China). The analysis was conducted separately on up- and down-regulated genes. For each group, a hypergeometric test was performed to identify statistically-significant enriched GO categories (False Discovery Rate (FDR) < 0.05, after Benjamini–Hochberg–Yekutieli procedure, BH, correction of the *p*-value). The enrichment score was calculated as: (query item/query total)/(BG item/BG total). Quantitative real-time PCR was performed for differential expression validation.

### 2.6. Oxylipins Assay

Extraction and quantitative analysis was performed as described in Scala *et al.* [21] and Ludovici *et al.* [22], respectively. Briefly, a rapid resolution reversed-phase HPLC (RR-RP-HPLC) separation followed by MS/MS detection with a triple quadrupole (QqQ) mass-spectrometer (Agilent Technologies, Santa Clara, CA, USA; G6420A) with an ESI interface was used. Multiple reaction monitoring (MRM) was adopted for the quantitative assessments of target oxylipins as reported in Ludovici *et al.* [22]. Concentrations were expressed as μM of compounds in the 200 μL of methanol volume used to dissolve the dry lipid extract.

## 3. Results

### 3.1. Physiological Responses to Menadione-Related Oxidative Stress

**Figure 1:**
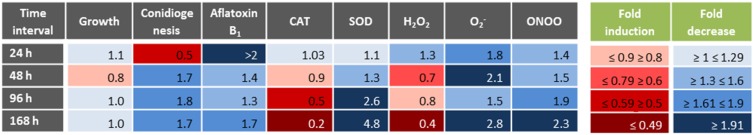
Fold induction/decrease of menadione (Men)-treated *vs.* untreated samples pinpointed in a color scale. The fungal growth (mg/mL), conidiogenesis (number of conidia/mL), aflatoxin B_1_ production (ppb), catalases (CAT and superoxide dismutases (SOD) activities (U/mg proteins), hydrogen peroxide (H_2_O_2_), superoxide anion (O_2_^−^) and peroxynitrite (ONOO) production (μM) were calculated for both Men-treated and untreated samples at different time intervals (24–168 h post-amendment (hpa)) of growth into PDB at 30 °C in dark conditions. Values represent the average of six replicates originated by two biological and three technical replications each ±SE.

Some physiological parameters, namely fungal growth, conidiogenesis, aflatoxin synthesis, ROS and antioxidant enzyme production, were evaluated in liquid culture of *A. flavus* 3357 amended with the oxidizer menadione (Men) at 0.1 mM at different time intervals (0–168 hpa). The results are presented in a composite table where the fold induction/decrease of Men-treated *vs.* untreated samples are pinpointed in a color scale (Figure 1). The complete list of results is provided into Supplementary File 1.

### 3.2. Correlations

**Figure 2:**
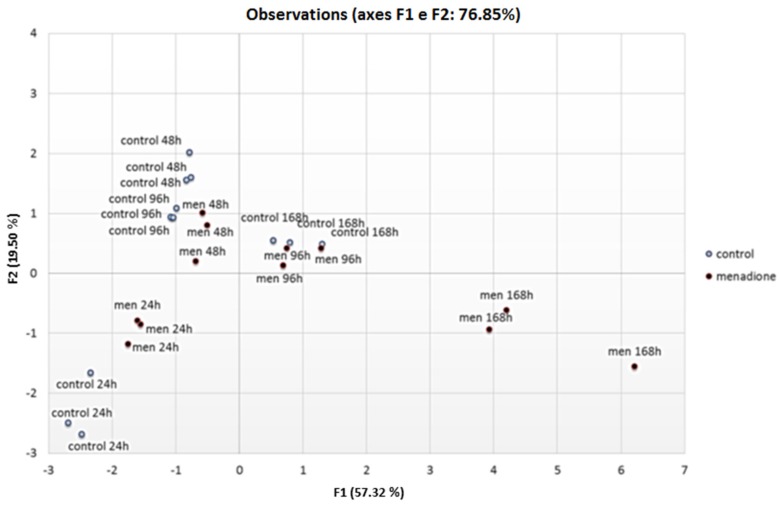
PCA score plot of data generated by analysis of several physiological parameters, such as fungal growth, conidiogenesis, aflatoxin B_1_ production, catalases and superoxide dismutases activities, hydrogen peroxide, superoxide anion and peroxynitrite production. Observations were clustered according to the sampling time (24–168 hpa) and to the treatment with menadione (Men) or its absence (control).

Most parameters showed a significant time-dependent increasing rate (Pearson correlation; see Table S2), and Men triggered some parameters, such as conidiogenesis, AFB_1_ synthesis, superoxide dismutase, superoxide anion and peroxynitrite production (Figure 1). Notably, in relation to AFB1 synthesis emerged a clear positive correlation with the ONOO^−^ amount (0.92) and a sharply negative correlation (−0.94) with anion superoxide. These data suggest a multifaceted impact of Men on *A. flavus* metabolism. To evaluate and validate if these physiological parameters are correlated in a time-dependent manner and if the difference in the time trend emerges between Men-treated and -untreated samples, we have performed a PCA. This analysis was performed on the whole dataset and showed a time-dependent grouping of the observations. Specifically, Men-treated samples at 48 and 96 hpa grouped with the 96 and 168 hpa of the control, respectively (Figure 2)*.* This could suggest that menadione speeds up the metabolic/growth rate of *A. flavus*, the parameters observed being related, directly and indirectly, to cell ageing (ROS, RNS antioxidant enzymes), metabolism (aflatoxin) and differentiation (growth, conidiogenesis).

### 3.3. Transcriptional Analysis of A. flavus Challenged with Menadione

**Figure 3:**
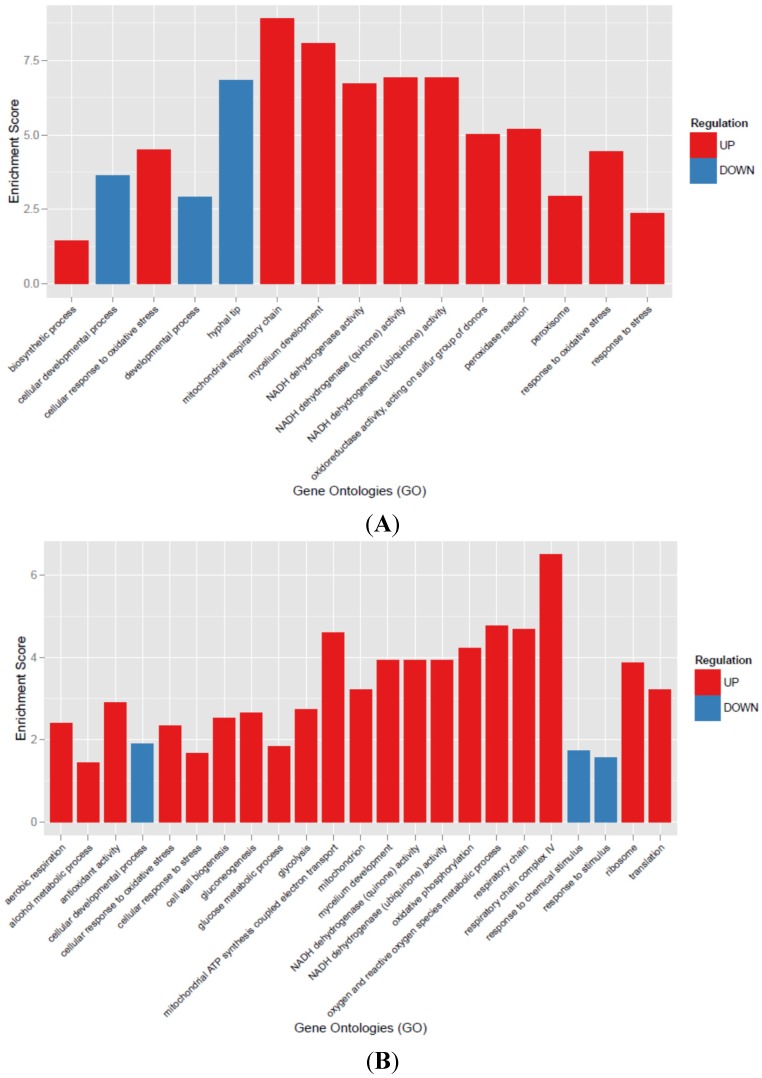

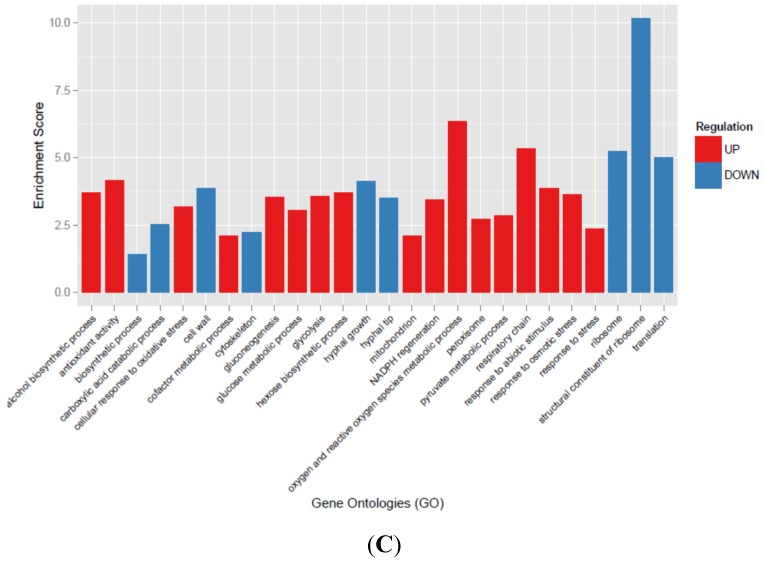
(**A**–**C**). Results of the GO enrichment analysis at 24 (**A**), 48 (**B**) and 96 (**C**) hpa. The *x*-axis reports the significantly-enriched categories identified among up- (red) and down- (blue) regulated genes after hypergeometric test (*p*-value < 0.01). The *y*-axis reports the enrichment score calculated as follows: (number of Differentially Expressed, DE, genes in category X/total number of DE genes)/(number of genes in the genome in category X/total number of genes in the genome).

The RNA-seq analysis was performed on samples at three time intervals (24–48–96 hpa) treated and untreated with Men 0.1 mM. Overall, 185 M raw reads were produced from the samples. After trimming and quality control, about 175 M of high quality reads with an average of 98.52% of reads mapped to the *A. flavus* genome. Details for each sample can be found in the Supplementary File 2. Differentially-expressed genes were analyzed to identify enriched functional categories by using GO annotations. Gene ontology annotation of *A. flavus* genes was downloaded from the AspGD database; about 10,000 genes had a GO annotation, and the total number of GO categories was 3677. Gene ontology enrichment analysis (GOEA) was performed on up- and down-regulated genes separately, and the significance of the enrichment was calculated by a hypergeometric-test (see the Materials and Methods for details). The complete results of the pathway analysis are presented in the Supporting Information (Supplementary Files 3), whereas in Figure 3A–C, the significantly-enriched (*p* < 0.01) categories are presented comparing Men-treated *vs.* untreated conditions in three different time intervals (24–48–96 hpa).

**Figure 4:**
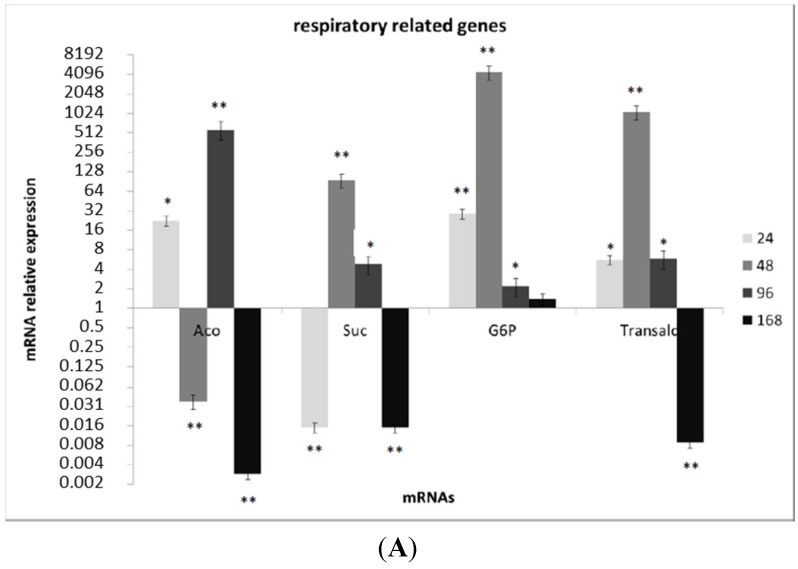

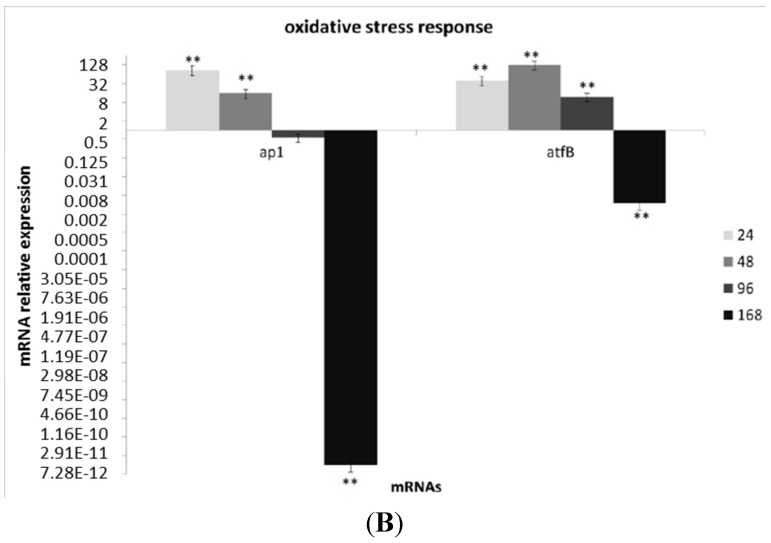
(**A**,**B**) Gene expressions evaluated by the SYBR-green qRT-PCR assay. The housekeeping gene to normalize target gene expression was beta-tubulin (Accession Number AFLA_051840). Relative expression (presented in log2 scale) was calculated by the 2^−ΔΔC*t*^ method considering the Men non-treated samples as calibrators in each time interval. The target genes considered were clustered according to their gene ontology, in cell respiration-related genes (aconitase, succinate dehydrogenase, glucose-6-*P*-dehydrogenase and transaldolase) and in oxidative stress response genes (*ap-1* and *atfB*). Values represent the average of six replicates originated by two biological and three technical replications each ±SE; * *p* < 0.05; ** *p* < 0.01 according to the Mann–Whitney test.

A subset of genes identified as differentially-expressed on RNA-seq were also verified by qRT-PCR (Figure 4A,B). Notably, we tested the expression of a subset of respiratory-related genes (aconitase, succinate dehydrogenase, glucose-6-*P*-dehydrogenase, transaldolase) and of transcription factors related to the response to oxidative stress (*AP-1* and *AtfB*). qRT-PCR results confirmed the upregulation at early and mid-times (24–48 hpa) of these subsets of genes indicated by RNA-seq analysis (Figure 3A,B).

### 3.4. Oxylipin Analysis of A. flavus Treated with Menadione

**Figure 5:**
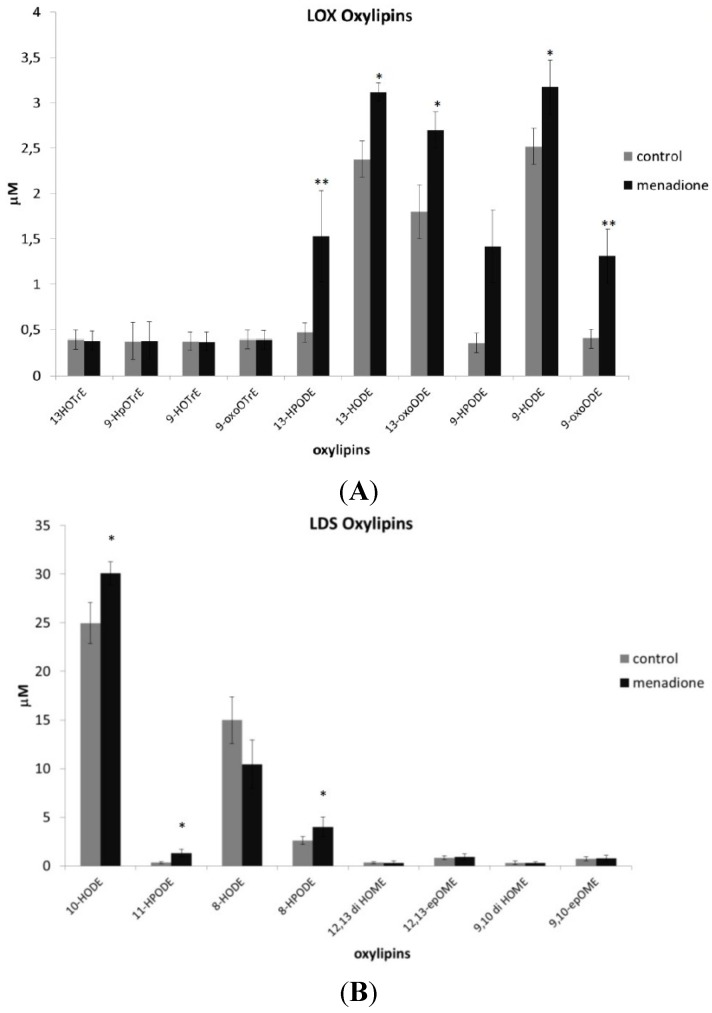
(**A**,**B**) Quantification of oxylipins under *in vitro* experiment. Quantitative LC-MS/MRM analysis of 19 oxylipins (μM) in mycelia of *A. flavus* treated (menadione) and untreated (control) with Men 0.1 mM, 96 hpa. LOX-oxylipins detected were 9 and 13 hydroxyoctadecatrienoic acid (HOTrE), 9-hydroperoxyoctadecatrienoic acid (HpOTrE), 9-oxo-octadecatrienoic acid (oxoOTrE), 9- and 13-hydroperoxyoctadecadienoic acid (HpODE), 9- and 13-hydroxyoctadecadienoic acid (HODE), 9- and 13-oxo-octadecadienoic acid (oxoODE). LDS-derived oxylipins detected were 8- and 10-hydroxyoctadecadienoic acid (HODE), 8- and 11-hydroperoxyoctadecadienoic acid (HpODE), 9,10- and 12,13-epoxyoctadecenoic acid (epOME), 9,10 and 12,13-dihydroxyoctadecenoic acid (di HOME). Values represent the average of six replicates originated by two biological and three technical replications each ±SE; * *p* < 0.05; ** *p* < 0.01 according to the Mann–Whitney test. LOX, lipoxygenases.

Since the RNA-seq analysis indicated the involvement of FA metabolism at later time intervals (96 hpa; Figure 3C), we analyzed the oxylipin content in the mycelia of *A. flavus* treated and untreated with menadione at a fixed time interval, *i.e.*, at 96 hpa (Figure 5A,B). The activation of the FA metabolism into the Men-treated mycelia apparently occurred in the same time interval (Figure 3C). The oxylipin set was divided according to its putative enzymatic origin, specifically, if linoleate synthases (LDS)- or lipoxygenases (LOX)-derived, following a published protocol [23]. LDS-related oxylipins are produced in higher amounts compared to the LOX-related ones. This is expected, since LDS is present in multiple copies (four) compared to the single copy of LOX present in the *A. flavus* 3357 genome. Nevertheless, Men treatment affected less LDS-related oxylipins than the LOX-related ones, which conversely changed significantly upon oxidizer amendment (Figure 5A,B). Notably, extracting gene expression from the RNA-seq data (Supplementary File 4) resulted in the upregulation of LOX for the whole time course (24–96 hpa; 4.7, 3.5 and 2.9, respectively) of the experiment consequent to Men amendment.

## 4. Discussion

The ecological role of aflatoxin is still debated. An intriguing hypothesis is the one derived from research performed in the last ten years by various groups [3,24,25,26]. Aflatoxin production apparently directs the excess of oxidants accumulated during cell ageing, or under external insults, into extending the lifespan of the fungus [26]. To further confirm the correlation between oxidative stress and aflatoxin synthesis, we treat *A. flavus* with an oxidizing agent, *i.e.*, menadione. Menadione, once in the cell, may release anion superoxide [12]. The superoxide anion produced could alternatively be scavenged by superoxide dismutases (SOD), being transformed into hydrogen peroxide, or react with nitric oxide to form peroxynitrite, a compound that plays an important role in the reaction of fungi to xenobiotic stress [27]. For some fungi, especially the pathogenic ones, experiencing transient or chronic oxidative stress conditions represents “normality”. In relation to this, managing reactive species for driving growth, metabolism and differentiation could transform a “foe” into a “friend”, *i.e.*, a menace into an opportunity to exploit new substrates or invading host tissues.

How may ROS re-program fungal cells? The most direct way is through oxidative stress-related transcription factors, such as the homologues of AP-1, AtfB, Hsf-2, Skn-7 and Msn2-4 [2,3,15,28]. These factors perceive reactive species; by posttranslational modifications, they can activate and start transcriptional responses by recognizing specific regulatory elements (RE), such as Yap Responsive Elements, YRE, and Antioxidant Responsive Elements, ARE. In some cases, these REs can be found in promoters of non-obvious genes, such as *AflR*, which is not directly involved in the antioxidant response, but in the regulation of the aflatoxin gene cluster [15,28]. This could mean that factors, such as *AP-1*, could control other aspects of fungal “life” beyond those of antioxidant defenses. For instance, the formation of the AP-1/AtfB heterodimer creates *de facto* a close link among energy utilization/metabolism/fungal growth. Moreover, chronic oxidative stress favors, probably at the peroxisomal level, the formation of peroxynitrite, starting from anion superoxide, and of nitric oxide accumulation [29,30]. This reactive non-radical species is implicated in several diseases in humans and plants, whilst its role in fungi is poorly described.

Our hypothesis is that chronic stimulation of ROS by Men would favor the production of these species that, in turn, could trigger lipid peroxidation, as affirmed elsewhere [31]. Since some authors indicate peroxisomes as the major source of peroxynitrite, it could be reasonable that this reactive species might prompt a spontaneous, non-enzymatic lipid peroxidation, probably followed by an enzymatic one. Peroxisomes in *A. flavus* are involved in the modulation of early oxidative stress onset, as well as to later (compared to growth phases) activation of FA metabolism [32]. The activation of FA metabolism could prompt the production of oxylipins, molecules crucial in driving fungal growth, metabolism and differentiation [32,33]. An organized network of signaling cascades that synchronize oxylipin products occurred in fungal mycelia as a response to different environmental conditions [34,35,36]. This fact could lead to an “oxylipins signature profile” typical for every pathogen [34]. Oxylipins in fungi may act as hormone-like substances [33]. Accordingly, some of the molecular and physiological events related to menadione addition could be ascribed to the stimulation of a specific type of oxylipin. Intriguingly, the most abundant products found in our study, *i.e.*, the LDS-related oxylipins, resulted in being less affected by Men treatment than the LOX-related ones. It is reported that mycotoxigenic fungi, such as *A. flavus* or *F. verticillioides*, possess more than one copy of LDS, but generally, one or two copies of LOX [36,37,38,39,40]. We can suggest that LDS-related oxylipins may act as “housekeeping” signals, whereas LOX-oxylipins could be more “spendable” for promptly reacting to external insult. This fact could partly explain the difference in the abundance of the relative oxylipins. Nevertheless, the trigger posed by Men on LOX-derived oxylipins emphasizes their putative role in modulating aflatoxin synthesis and conidiogenesis in *A. flavus*, as extensively stated elsewhere [38,39], even if LDS-derived ones could also support fungal reproduction, virulence and secondary metabolism [36]. Our results suggest that Men, even by accelerating cell metabolism and shortening the transition from tropho- to idio-phase, could foster, enzymatically or non-enzymatically, the production of LOX-derived oxylipins. In turn, these reactive molecules may start a signal cascade that, by an autocrine GPCR-mediated mechanism [41] or by chromatin remodeling [36], reshapes the fungal transcriptome and leads to a specific phenotype: mycotoxin production and conidiogenesis.

In conclusion, oxidative stress is confirmed as a pre-requisite for “modelling” fungal lifestyle also by fine-tuning the secondary metabolism. In relation to this, keeping the fungus in a perennial “juvenile” stage could aid its control regarding aflatoxin production. Hence, the use of antioxidants (even of “natural” origin) represent a concrete tool for limiting aflatoxin production in stored feed and foodstuff.

## Supplementary Materials

Supplementary materials can be accessed at: http://www.mdpi.com/2072-6651/7/10/4315/s1.

## References

[1] Jayashree T., Subramanyam C. (2000). Oxidative stress as a prerequisite for aflatoxin production by *Aspergillus parasiticus*. Free Radic. Biol. Med..

[2] Reverberi M., Fabbri A.A., Zjalic S., Ricelli A., Punelli F., Fanelli C. (2005). Antioxidant enzymes stimulation in *Aspergillus parasiticus* by *Lentinula edodes* inhibits aflatoxin production. Appl. Microbiol. Biotechnol..

[3] Reverberi M., Zjalic S., Ricelli A., Punelli F., Camera E., Fabbri C., Picardo M., Fanelli C., Fabbri A.A. (2008). Modulation of antioxidant defence in *Aspergillus parasiticus* is involved in aflatoxin biosynthesis: A role for the ApyapA gene. Eukaryot. Cell.

[4] Schrader M., Fahimi H.D. (2006). Peroxisomes and oxidative stress. Biochim. Biophys. Acta Mol. Cell Res..

[5] Halliwell B. (2006). Reactive species and antioxidants. Redox biology is a fundamental theme of aerobic life. Plant Physiol..

[6] Mittler R., Vanderauwera S., Suzuki N., Miller G., Tognetti V.B., Vandepoele K., Gollery M., Shulaev V., van Breusegem F. (2011). ROS signalling: The new wave?. Trends Plant Sci..

[7] Apel K., Hirt H. (2004). Reactive oxygen species: Metabolism, oxidative stress, and signal transduction. Annu. Rev. Plant Biol..

[8] Dowling D., Simmons L.W. (2009). Reactive oxygen species as universal constraints in life-history evolution. Proc. R. Soc. B.

[9] Apostol I., Heinstein P.F., Low P.S. (1989). Rapid stimulation of an oxidative burst during elicitation of cultured plant cells. Role in defense and signal transduction. Plant Physiol..

[10] Thorpe G.W., Fong C.S., Alic N., Higgins V.J., Dawes I.W. (2004). Cells have distinct mechanisms to maintain protection against different reactive oxygen species: Oxidative-stress-response genes. Proc. Natl. Acad. Sci. USA.

[11] Monks T.J., Hanzlik R.P., Cohen G.M., Ross D., Graham D.G. (1992). Quinone chemistry and toxicity. Toxicol. Appl. Pharmacol..

[12] Criddle D.N., Gillies S., Baumgartner-Wilson H.K., Jaffar M., Chinje E.C., Passmore S., Chvanov M., Barrow S., Gerasimenko O.V., Tepikin A.V. (2006). Menadione-induced reactive oxygen species generation via redox cycling promotes apoptosis of murine pancreatic acinar cells. J. Biol. Chem..

[13] Calvo A.M., Wilson R.A., Bok J.W., Keller N.P. (2002). Relationship between secondary metabolism and fungal development. Microbiol. Mol. Biol. Rev..

[14] Bonekamp N.A., Völkl A., Fahimi H.D., Schrader M. (2009). Reactive oxygen species and peroxisomes: Struggling for balance. BioFactors.

[15] Reverberi M., Zjalic S., Punelli F., Ricelli A., Fabbri A.A., Fanelli C. (2007). Apyap1 affects aflatoxin biosynthesis during *Aspergillus parasiticus* growth in maize seeds. Food Addit. Contam..

[16] Cao Q.H., Zhou Q.X., Cai R.X., Liu Z.H. (2005). Fluorimetric determination of peroxynitrite based on an enzymatic reaction. Anal. Sci..

[17] Bolger A.M., Lohse M., Usadel B. (2014). Trimmomatic: A flexible trimmer for Illumina Sequence Data. Bioinformatics.

[18] Kim D., Pertea G., Trapnell C., Pimentel H., Kelley R., Salzberg S.L. (2013). TopHat2: Accurate alignment of transcriptomes in the presence of insertions, deletions and gene fusions. Genome Biol..

[19] Liao Y., Smyth G.K., Shi W. (2014). Feature Counts: An efficient general-purpose program for assigning sequence reads to genomic features. Bioinformatics.

[20] Robinson M.D., Davis J., McCarthy D., Gordon K.S. (2010). edgeR: A Bioconductor package for differential expression analysis of digital gene expression data. Bioinformatics.

[21] Scala V., Camera E., Ludovici M., Dall’Asta C., Cirlini M., Giorni P., Battilani P., Bello C., Fabbri A.A., Fanelli C. (2013). *Fusarium verticillioides* and maize interaction *in vitro*: Relation between oxylipin cross-talk and fumonisin synthesis. World Mycotoxin J..

[22] Ludovici M., Ialongo C., Reverberi M., Beccaccioli M., Scarpari M., Scala V. (2014). Quantitative profiling of oxylipins through comprehensive LC-MS/MS analysis of *Fusarium verticillioides* and maize kernels. Food Addit. Contam. A.

[23] Strassburg K., Huijbrechts A.M., Kortekaas K.A., Lindeman J.H., Pedersen T.L., Dane A. (2012). Quantitative profiling of oxylipins through comprehensive LC-MS/MS analysis: Application in cardiac surgery. Anal. Bioanal. Chem..

[24] Narasaiah K.W., Sashidhar R.B., Subramanyam C. (2006). Biochemical analysis of oxidative stress in the production of aflatoxin and its precursor intermediates. Mycopathologia.

[25] Reverberi M., Punelli M., Scala V., Scarpari M., Uva P., Mentzen W.I., Dolezal A.L., Woloshuk C., Pinzari F., Fabbri A.A. (2013). Genotypic and phenotypic versatility of *Aspergillus flavus* during maize exploitation. PLoS ONE.

[26] Hong S.Y., Roze L.V., Linz J.E. (2013). Oxidative stress-related transcription factors in the regulation of secondary metabolism. Toxins.

[27] Ferreira G.F., Baltazar Lde M., Santos J.R., Monteiro A.S., Fraga L.A., Resende-Stoianoff M.A., Santos D.A. (2013). The role of oxidative and nitrosative bursts caused by azoles and amphotericin B against the fungal pathogen *Cryptococcus gattii*. J. Antimicrob. Chemother..

[28] Roze L.V., Chanda A., Wee J., Awad D., Linz J.E. (2011). Stress-related transcription factor AtfB integrates secondary metabolism with oxidative stress response in *Aspergilli*. J. Biol. Chem..

[29] Szabo C., Ischiropoulos H., Radi R. (2007). Peroxynitrite: Biochemistry, pathophysiology and development of therapeutics. Nat. Rev. Drug Discov..

[30] Virag L., Szabo E., Gergely P. (2003). Peroxynitrite-induced cytotoxicity: Mechanism and opportunities for intervention. Toxicol. Lett..

[31] Rubbo H., Trostchansky A., O’Donnell V.B. (2009). Peroxynitrite-mediated lipid oxidation and nitration: Mechanisms and consequences. Arch. Biochem. Biophys..

[32] Reverberi M., Punelli M., Smith C.A., Zjalic S., Scarpari M., Scala V., Fanelli C. (2012). How peroxisomes affect aflatoxin biosynthesis in *Aspergillus flavus*. PLoS ONE.

[33] Tsitsigiannis D.I., Keller N.P. (2007). Oxylipins as developmental and host-fungal communication signals. Trends Microbiol..

[34] Reverberi M., Fabbri A.A., Fanelli C., Guenther W. (2012). Oxidative stress and oxylipins in plant-fungus interaction. Biocommunication of Fungi.

[35] Reverberi M., Punelli F., Scarpari M., Camera E., Zjalic S., Ricelli A., Fanelli C., Fabbri A.A. (2010). Lipoperoxidation affects ochratoxin A biosynthesis in *Aspergillus ochraceus* and its interaction with wheat seeds. Appl. Microbiol. Biotechnol..

[36] Scala V., Giorni P., Cirlini M., Ludovici M., Visentin I., Cardinale F., Fabbri A.A., Fanelli C., Reverberi M., Battilani P. (2014). LDS1-produced oxylipins are negative regulators of growth, conidiation and fumonisin synthesis in the fungal maize pathogen *Fusarium verticillioides*. Front. Microbiol..

[37] Tsitsigiannis D.I., Kowieski R., Zarnowski R., Keller N.P. (2005). Three putative oxylipin biosynthetic genes integrate sexual and asexual development in *Aspergillus nidulans*. Microbiology.

[38] Brown S., Zarnowski R., Sharpee W.C., Keller N.P. (2008). Morphological transitions governed by density dependence and lipoxygenase activity in *Aspergillus flavus*. Appl. Environ. Microbiol..

[39] Scarpari M., Punelli M., Scala V., Zaccaria M., Nobili C., Ludovici M., Camera E., Fabbri A.A., Reverberi M., Fanelli C. (2014). Lipids in *Aspergillus flavus*-maize interaction. Front. Microbiol..

[40] Scala V., Beccaccioli M., Dall’Asta C., Giorni P., Fanelli C. (2015). Analysis of the expression of genes related to oxylipin biosynthesis in *Fusarium verticillioides* and maize kernels during their interaction. J. Plant Pathol..

[41] Affeldt K.J., Brodhagen M., Keller N.P. (2012). *Aspergillus* oxylipin signalling and quorum sensing pathways depend on G Protein-Coupled Receptors. Toxins.

