# Supplementary material for: Menadione-Induced Oxidative Stress Re-Shapes the Oxylipin Profile of Aspergillus flavus and Its Lifestyle
Source: Toxins (Basel). 2015 Oct 23;7(10):4315–29. doi: 10.3390/toxins7104315 (PMC4626736; doi:10.3390/toxins7104315)
Supplement: Supplementary file 1 [file toxins-07-04315-s001.zip › toxins-94836-supplementary materials/toxins-94836-supplementary table S1-table S2_rvz.pdf]

Supplementary Materials

Table S1. Primer sequences.

Gene	Primer FOR	Primer REV
**Aconitase**	5′-GCGACAAGGAAGGGTGTTTT-3′	5′-GGAAAGCAGTCTCCCAGAGT-3′
**Succinate dehydrogenase**	5′-TTCTTCTGCCACGTGAGAGT 3′	5′-TCTTTTGACCTGCGCGTATG-3′
**G-6-p dehydrogenase**	5′-TTCGGGGATCTTAAGGTCGG-3′	5′-ACTTCGGGCATCTTCAAGGA-3′
**Transaldolase**	5′-TTTCCGGGGATGATCTCGAG-3′	5′-CAGCACGTACGTCATCCTTG-3′
AP-1	5′-GGTTGTTTGAGCCGTTGAGT 3′	5′-ACGGCCTCAATAACAACGAC-3′
AtfB	5′-CACCTGCGCAGCGAAGT-3′	5′-CGCATGCCGCAGCAT-3′

Table S2. Pearson correlations among different parameters [fungal growth, conidiogenesis, AFB_1 synthesis, superoxide dismutase (SOD), catalase (CAT), hydrogen peroxide (H_2O_2), superoxide anion (ASO) and peroxynitrite production (ONOO)] expressed under (**A**) control conditions and (**B**) treatment with 0.1 mM of menadione by *A. flavus* at different time intervals of observation (0–168 hpa).

(A)

**Variables [1]**	**Growth**	**Conidiogenesis**	**AFB1**	**CAT**	**SOD**	**H_2O_2**	**ASO**	**ONOO**
Time	**0.609654**	**0.908782117**	**0.768303**	−0.37456	0.465938	−0.36338	**−0.82038**	**0.692778**
growth	-	0.253747248	**0.911778**	0.26252	0.231837	0.407772	**−0.84**	**0.947715**
Conidiogenesis	-	-	0.439646	**−0.66234**	0.555358	**−0.69881**	**−0.6073**	0.37122
AFB1	-	-	-	0.247248	0.093194	0.263343	**−0.7989**	**0.902429**
CAT	-	-	-	-	**−0.77547**	**0.83112**	0.238899	0.107151
SOD	-	-	-	-	-	−0.55248	**−0.64755**	0.363224
H2O2	-	-	-	-	-	-	0.064574	0.244587
ASO	-	-	-	-	-	-	-	**−0.90378**

(B)

**Variables [1]**	**Growth**	**Conidiogenesis**	**AFB1**	**CAT**	**SOD**	**H_2O_2**	**ASO**	**ONOO**
Time	**0.924199**	0.571149662	**0.959767**	**−0.94673**	**0.937318**	**−0.84207**	**−0.81551**	**0.948643**
growth	-	0.574247711	**0.946545**	**−0.83795**	**0.797845**	**−0.65452**	**−0.89407**	**0.940706**
Conidiogenesis	-	-	0.553489	−0.54048	**0.639314**	**−0.62097**	−0.4491	**0.680415**
AFB1	-	-	-	**−0.84291**	**0.890135**	**−0.75651**	**−0.93845**	**0.91803**
CAT	-	-	-	-	**−0.85763**	**0.792345**	**0.631716**	**−0.90906**
SOD	-		-	-	-	**−0.94395**	**−0.71512**	**0.891644**
H_2O_2	-	-	-	-	-	-	0.552172	**−0.76677**
ASO	-	-	-	-	-	-	-	**−0.77261**

[1] Statistic significance: bold values are different from 0 at the significance level of $\alpha = 0.05$.
